# Interaction of Thioamides, Selenoamides, and Amides With Diiodine

**DOI:** 10.1155/BCA/2006/60291

**Published:** 2006-12-13

**Authors:** Sotiris K. Hadjikakou, Nick Hadjiliadis

**Affiliations:** Section of Inorganic and Analytical Chemistry, Department of Chemistry, University of Ioannina, 45110 Ioannina, Greece

## Abstract

We review the results of our work on the iodine interaction with thioamides, selenoamides, and amides. Complexes with (i) “spoke” or “extended spoke” structures, D · I_2_ and D · I_2_ · I_2_, respectively, (D is the ligand donor) (ii) iodonium salts of {[D_2_ − I]^+^[I_*n*_]^−^} (*n* = 3, 7) and {[D_2_ − I]^+^[FeCl_4_]^−^} formulae and (iii) disulfides of the categories (a) [D − D], (b) {[D − DH]^+^[I_3_]^−^} have been isolated and characterized. A compound of formula {[D_2_ − I]^+^[I_3_]^−^[D · I_2_]} containing both types of complexes (i) and (ii) was also isolated. The interaction of diiodine with selenium analogs of the antithyroid drug 6-*n*-propyl-2-thiouracil (PTU), of formulae RSeU (6-alkyl-2-Selenouracil) results in the formation of complexes with formulae [(RSeU)I_2_]. All these results are correlated with the mechanism of action of antithyroid drugs. Finally, we review here our work on the diiodine interaction with the amides (LO).

## INTRODUCTION

The perturbation of the I−I bond when diiodine binds to heterocycles such as thioamides or selonoamides results to novel complexes containing iodine [1–13]. Various types of such complexes have been obtained thus far, including charge transfer (ct) complexes with the so-called “spoke” or “extended spoke” structures (DS · I_2_ or DS · I_2_ · I_2_) (D is the ligand donor) [6], the “T-shape” structures, iodine(I) coordinated to two thioamides to form “iodonium salts” ([DS−I−DS]^+^ · (I_3_)^−^) [6], oxidation products of formulae (DS−I)^+^ [6], dications of formulae ([DS−SD]^2+^(I_*n*_^−^)_2_), [6] and monocations of formulae ([DS−SDH]^+^(I_3_)^−^) [6] (DS or D=S, a thioamide ligand donor).

The interest in studying I_2_ interaction with thioamides and selenoamides arises from their application in both biological chemistry and material sciences. Thus,

- thioamides, like 6-*n*-propyl-thiouracil (PTU), N-methyl-imidazoline-2-thione (methimazole, MMI), 3-methyl-2-thioxo-4-imidazoline-1-carboxylate (carbimazole) (CBZ), are known antithyroid drugs [14] against hyperthyroidism, while thiazolidine-2-thione (TZD) and 1,3-bis(hydroxymethyl)-benzimidazoline-2-thione (BHBZIM) were also used as such, in the past. Hyperthyroidism (Grave's disease) is characterized by the overproduction of *T*4 and *T*3 hormones. The way that the above and other similar thioamides interact with I_2_ is of great importance, in an attempt to approximate the elucidation of their mechanism of action, since I_2_ is involved in the synthesis of both *T*4 and *T*3 hormones [15–17];
- iodine chemistry is recently proving to be of considerable interest because of the discovery of low-temperature, semi- and superconducting polyiodides, which quickly led to the deliberate doping of conjugated polymers with elemental iodine [13]. The ability of iodine to catenate leads to the formation of polyiodides of various structures [13] reviewed recently by Boyle et al, Deplano et al, and Svensson et al [10–13]. The structural variety of polyiodides ranges from the simple I^−^_3_ through the linear I^2−^_4_, the V-shaped I^−^_5_, three-pronged structures of I^−^_7_, I^−^_9_, I^2−^_10_, and I^2−^_12_, the Z-shaped I^−^_7_, I^2−^_8_, the branched I^2−^_16_, and the S-shaped and linear I^4−^_16_ species to infinite chains [5]. The size and shape of the polyiodide ions have been found to depend in an unpredictable way on the size and shape of the counter ion.

In this paper, we review the results of our work on the iodine interaction with thioamides. Complexes of the so-called “spoke” and “extended spoke” structures, D · I_2_ and D · I_2_ · I_2_, respectively, (D is the ligand donor) (ii) iodonium salts of {[D_2_−I]^+^[I_*n*_]^−^} (*n* = 3, 7) and {[D_2_−I]^+^[FeCl_4_]^−^} formulae and (iii) disulfides of the categories (a) [D−D] and (b) {[D−DH]^+^[I_3_]^−^} produced by oxidation action of I_2_, have been isolated and characterized. A compound of formula {[D_2_−I]^+^[I_3_]^−^[D · I_2_]} containing both types of complexes (i) and (ii) was also isolated.

In addition the results on interaction between diiodine with selenium analogs of the antithyroid drug 6-*n*-propyl-2-thiouracil (PTU) of formulae RSeU have also been included. Complexes of formulae [(RSeU)I_2_] with “spoke” structures have been isolated. These complexes are stable in nonpolar solvents, but they decompose in polar solvents, producing diselenide compounds or undertaking deselenation. All these results are well correlated with the mechanism of action of antithyroid drugs. Finally, we review here our work on the diiodine interaction with the amides (LO) (L = organic framework), 2-hydroxy-pyridine and 2-hydroxy-pyrimidine. Complexes of formulae {(LO)_3_[(LO)]^+^ · I^−^_3_}, {(LO)_6_ · [(LO)_2_]^2+^ · ((1/2)I^−^) · ((3/2)I^−^_7_) · (I_2_)}, as well as {[LOH]^+^Cl^−^I_2_} have been isolated and characterized.

**Scheme 1:**
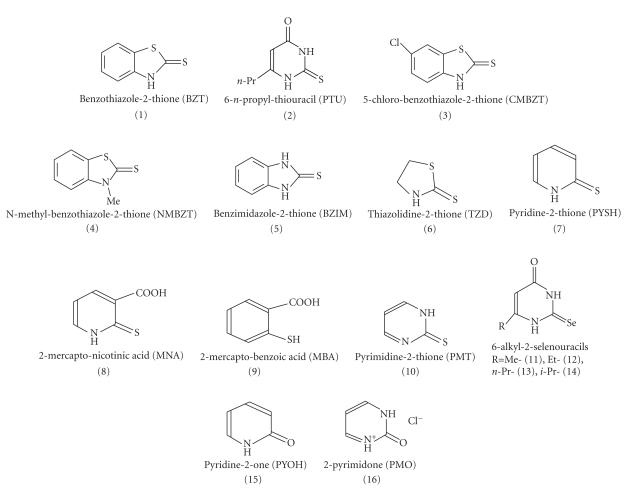
Ligands used in our work.

The ligands used in the present study are summarized in Scheme 1.

## RESULTS AND DISCUSSION

### Synthesis of thioamide-diiodine complexes

#### Charge transfer complexes with “spoke” or “extended spoke” structures

**Scheme 2:**
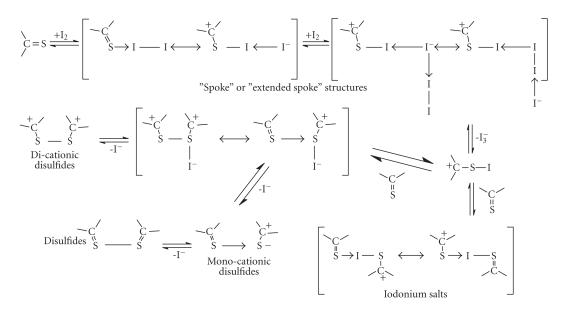


Reactions between diiodine and the thioamides (1)–(5) (Scheme 1) lead to the formation of charge transfer (ct) complexes with the so-called “spoke” or “extended spoke” structures (DS · I_2_ or DS · I_2_ ⋯ I_2_) according to the general reaction shown in Scheme 2.

**Scheme 3:**
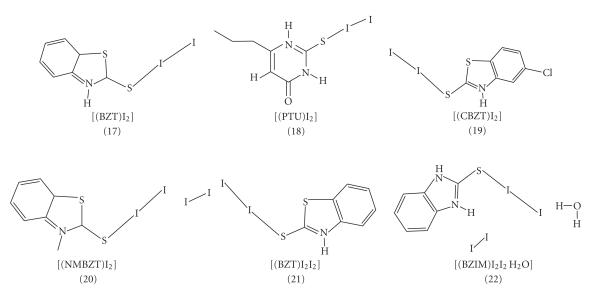
Molecular diagrams of ct complexes with spoke and extended spoke structures.

Thus, reaction of diiodine with (1), (2), (3), or (4) in a molar ratio 1 : 1 (I_2_ : L) results to the formation of ct complexes of formulae [(BZT)I_2_] (17) [1], [(PTU)I_2_] (18) [3], [(CBZT)I_2_] (19) [3], and [(NMBZT)I_2_] (20) [6] with spoke structures (Scheme 3). Reactions of diiodine with (1) or (5) in 2 : 1 (I_2_ : L) molar ratio form ct complexes of extended spoke structures with formulae [(BZT)I_2_I_2_] (21) [1] and [(BZIM)I_2_I_2_H_2_O] (22) [1] (Scheme 3).

Table 1 summarizes bond distances and angles of importance of our ct complexes with spoke and extended spoke structures.

The I−I bond distances are varying from 2.79 Å, in case of complexes with weak I−S interaction, to 3.08 Å, as a result of a strong I−S interaction. The corresponding I−I bond is subsequently elongated with respect to the corresponding distance in free I−I in the solid state [19] (2.717 Å at 110 K [20]). Bigoli et al [21] has classified iodine adducts of sulfur donors into three classes, depending on I−I bond order (*n*), calculated from Pauling's equation d(I−I) = d_0_ − 0.85 · log(*n*) (1) [where d is I−I interatomic distance of the adduct, d_0_ is the I−I bond distance of gas phase I_2_ (2.67 Å), and *n* is the I−I bond order] [18]. When *n* ≥ 0.6 and d(I−I) < 2.85 Å, the adduct is type A, and when *n* ≤ 0.4 and d(I−I) > 3.01 Å, it is type C. Compounds with intermediate values were classified as type B. Thus, compounds (18) and (20) are classified into A type, compounds (19), (21), and (22) into B type, whereas compound (17) is C type.

**Figure 1:**
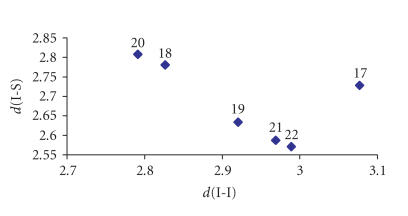
Correlation between d(I−S) and d(I−I) found for spoke and extended spoke ct complexes of diiodine, [(BZT)I_2_] (17), [(PTU)I_2_] (18), [(CBZT)I_2_] (19), [(NMBZT)I_2_] (20), [(BZT)I_2_I_2_] (21), and  [(BZIM)I_2_I_2_H_2_O] (22).

Figure 1 correlates d(I−I) versus d(I−S). A linear correlation is observed with the exception of complex (17). This behavior has been already explained [22].

The S−I−I group has a linear structure with an angle of almost 180°. The N−C−S−I torsion angle is also found almost equal to 180° indicating an almost coplanar arrangement of the I_2_ towards > C=S bond except the case of [(PTU)I_2_] complex, where it is found to be −95.93°. In fact, [(PTU)I_2_] complex is the first ct complex with perpendicular arrangement of I_2_ towards > C=S characterized by X-ray crystallography [3].

**Scheme 4:**
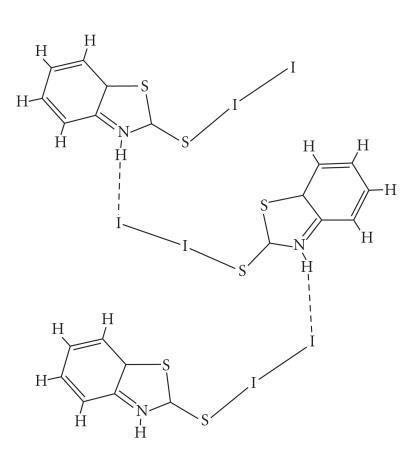
1D intermolecular network of complex [(BZT)I_2_].

An extended intermolecular 1D network through hydrogen bonding interaction is also formed in complex [(BZT)I_2_] (17) with N ⋯ I = 3.597(18) Å (Scheme 4).

#### Iodonium salt complexes

**Scheme 5:**
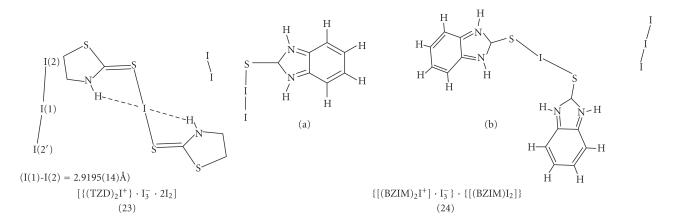


Reaction between diiodine and thioamides such as TZD (6) or BZIM (5) leads to the formation of iodonium salt complexes of [{(TZD)_2_I^+^}·I^−^_3_ · 2I_2_] (23) (Scheme 5) and {[(BZIM)_2_I^+^]I^−^_3_}{[(BZIM)I_2_]} (24) (Scheme 5) formulae according to the reaction in Scheme 2.

**Scheme 6:**
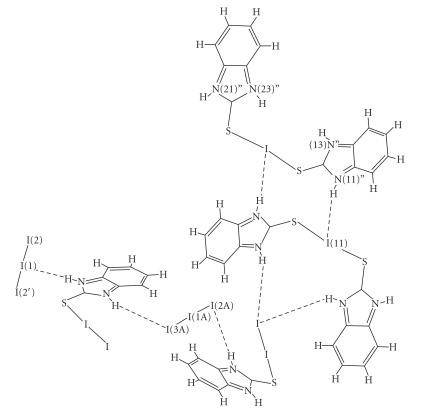
Hydrogen bonding interactions taking place in (24).

The two I−S bond distances are 2.654(6) Å in (23) and in case of (24) they are 2.597(4) Å and 2.702(4) Å, respectively. The I−I bond distances in I^−^_3_ counter anions are found to be equal (I−I = 2.9195(14) Å) in case of (23). In case of (24) two types of I^−^_3_ counter anions are observed, one is symmetric with I(1)−I(2) = 2.9300(12) Å while the other is not since it participates in hydrogen bonding (I(1A)−I(2A)= 2.880(6) Å and I(1A)−I(3A) = 3.058(5) Å, resp) and better described as I^−^, interacting with I_2_(I ⋯ I_2_) [11]. The two hydrogen bonding interactions are taking place between the I^+^ and the hydrogen atoms of the amide nitrogen atoms (H[N] ⋯ I = 2.9336(6) Å) in case of (23) (Scheme 5) and I(11) ⋯ H−N(11)″ of 3.20 Å in case of (24) (Scheme 6). *Ab initio* quantum mechanical methods and density functional theory (DFT) techniques applied on the iodonium part of (24) suggested that the conformations obtained in the crystalline state result from an intermolecular electrostatic interaction between the positively charged iodine and the negatively charged NH (total group (NH) charges calculated −0.005 e for N(11)″, −0.014 e for N(21)″, −0.004 e for N(13)″, and −0.012 e for N(23)″) [6] (Scheme 6).

**Scheme 7:**
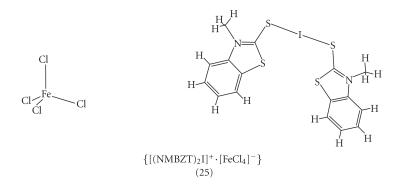
{[(NMBZT)_2_I]^+^· [FeCl_4_]^−^}(25).

When (NMBZT) (4) reacts with diiodine in the presence of FeCl_3_ in a molar ratio of 3 : 6 : 1 (NMBZT : I_2_ : FeCl_3_) (1), complex {[(NMBZT)_2_I]^+^ · [FeCl_4_]^−^} (25) (Scheme 7) together with {[(NMBZT)_2_I^+^]}·[I_7_]^−^ were formed [6]. The I−S bond distances are I−S = 2.5961(15) Å and 2.6596(14) Å, respectively, with an almost linear S−I^+^−S arrangement (S−I−S = 177.77(5)°)

$$\begin{array}{l} 3\text{NMBZT}+6{\text{I}}_{2}+{\text{FeCl}}_{3}\to \left(\frac{1}{2}\right)\left\{\left[{\left(\text{NMBZT}\right)}_{2}{\text{I}}^{+}{\left[{\text{FeCl}}_{\text{4}}\right]}^{-}\right]\right\}\\ +\left\{{\left[{\left(\text{NMBZT}\right)}_{2}{\text{I}}^{+}\right]}^{•}\left[{\text{I}}_{7}^{-}\right]\right\}+\text{unidentified products.} \end{array}$$ (1)

#### Monocationic and neutral disulfides

**Scheme 8:**
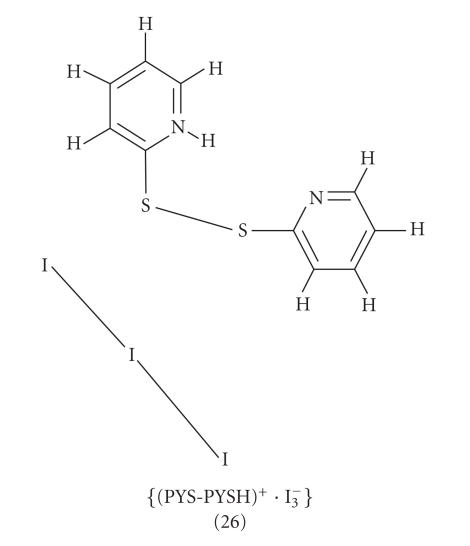
{(PYS-PYSH)^+^ · I^−^_3_}(26).

The reaction of 2-mercaptopyridine (PYSH) (7) with diiodine in a molar ratio of 1 : 2 led to the oxidation and dimerization of the ligand and produced {(PYS−PYSH)^+^ · I^−^_3_} (26) (Scheme 8). The structure of the compound consists of two residues; one cationic (PYS−PYSH)^+^, containing the S−S bond linking the two 2-mercapto-pyridine molecules one of which is protonated, and one I^−^_3_ counter anion. In the crystal lattice there are four symmetry-independent cation-anion pairs. There are only a few crystal structures reported in the literature containing open chain stable cations of DS-SD dimers, such as the monocationic: {[(C_4_H_6_N_2_S−SN_2_C_4_H_5_)_2_]^2+^ · (I^−^_3_) · (I^−^_5_)} [15]. The two I−I bond distances of the I^−^_3_ in the four components of complex (26) are 2.887(4) Å and 2.944(3) Å in component a, 2.874(4) Å and 2.957(3) Å in b, 2.968(3) Å and 2.862(3) Å in c, and 2.855(4) Å and 2.927(3) Å in d, respectively, indicating a slight asymmetry of I_3_^−^ in this complex (covalent linear asymmetric).

**Scheme 9:**
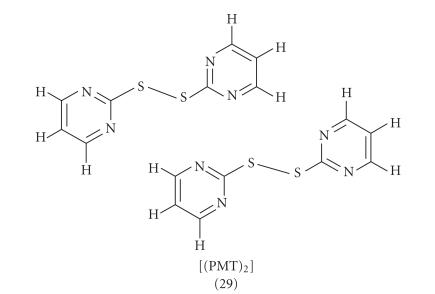
[(PMT)_2_](29).

Moreover, when MNA (8), MBA (9), or PMT (10) reacts with diiodine under the same experimental conditions as in the case of the preparation of complex (7), (see above), neutral disulfides were produced with formulae [(MNA)_2_] (27), [(MBA)_2_] (28), and [(PMT)_2_] (29) according to the reaction shown in Scheme 2. Scheme 9 shows the disulfide formed in case of (10) with formula [(PMT)_2_] (29).

**Scheme 10:**
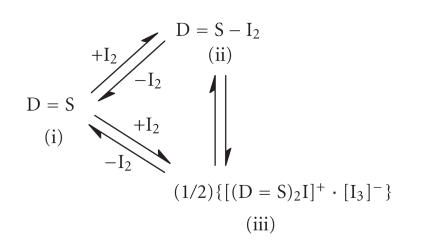


Complex (24) isolated from the reaction of (5) and I_2_ reveals the cocrystallization of both a “spoke” structure and an iodonium salt structure. This leads to the conclusion that the equilibrium of Scheme 10 is established in solution.

It has also been shown that the disproportionation reaction, with the generation of the ionic compound from thioamide-iodine complexes, exhibits pressure dependence [2]. A pressure increase leads to the ionic iodonium salt (iii) from (ii) (Scheme 10). The favoring of {[(MBZIM)_2_I]^+^[I_3_]^−^} (24a) formation is also proved by computational studies, based on energetic grounds [6].

**Scheme 11:**
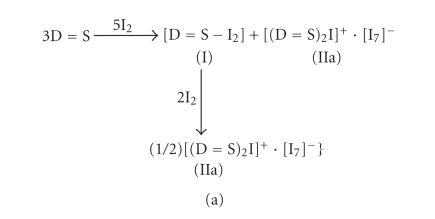

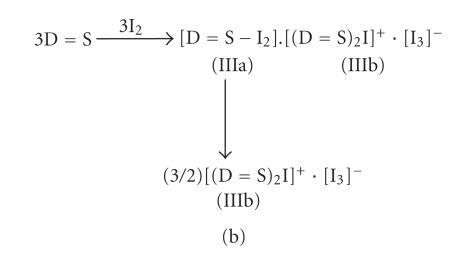


The conductivity measurements indicate that when diiodine is added to a solution of BZIM (5) (D=S), initially both the neutral (I) and the ionic (IIa) compounds are formed as it is shown in Scheme 11(a). Further addition of diiodine results to the ionic complex (IIa). In the case of NMBZT (Scheme 11(b)) a cocrystallization of both the spoke and iodonium complexes takes place producing only the iodonium complex, in excess of I_2_.

**Scheme 12:**
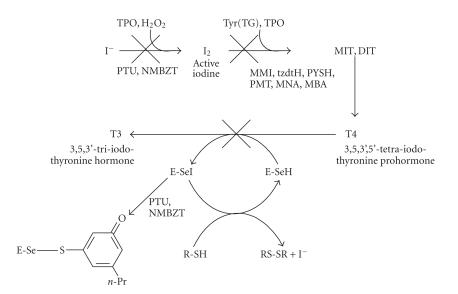


For the mechanism of action of antithyroid drugs the reaction scheme shown in Scheme 12 is followed.

Our results strongly indicate that the antithyroid drugs PTU (2) and N-Methyl-2-mercapto-imidazoline (MMI) have a different way of action. Thus, (2) together with NMBZT (4) forming weak S−I ct complexes (Table 1) may interfere either by inhibiting TPO activity [26] or by inhibiting deiodinase (ID-1) enzyme which is responsible for the formation of *T*3 from *T*4 hormone.

MMI, TZD (6), PYSH (7), PMT (10), MNA (8), and MBA (9), on the other hand, that strongly bind to I_2_ or are oxidized to disulfides [15] most probably interfere in the formation of monoiodotyrosine (MIT), diiodotyrosine (DIT) by the tyrosine residues of thyroglobulin Tyr(TG), competing with active iodine.

### Synthesis of selenoamide-diiodine complexes

**Scheme 13:**
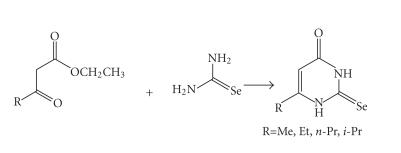
Synthesis of 6-alkyl-2-selenouracil ligands (11)−(14).

Since thyroid deiodinase contains selenocysteine [27], the seleno-analog of PTU (PSeU) is expected to exhibit a higher antithyroid activity than PTU, because of the easier formation of Enzyme-Se-Se-PSeU species than Enzyme-Se-S-PTU due to the higher nucleophilicity of Se. To examine this possibility we have extended our studies to the interaction of I_2_ with selenoamides. Scheme 1 shows the ligand used in this work. Ligands (11)–(14) were synthesized according the reactions shown in Scheme 13.

**Scheme 14:**
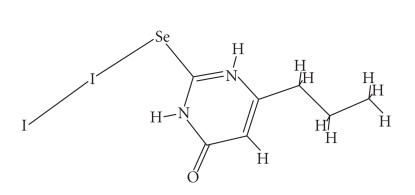
Molecular structure of [(*n*-PrSeU)I_2_](30).

Reactions of alkyl-selenoamides with diiodine in a 1 : 1 molar ratio in dichloromethane solutions result in the formation of [(RSeU)I_2_] [R = methyl-, ethyl-, *n*-propyl-, and *i*-propyl-] (Scheme 14).

**Figure 2:**
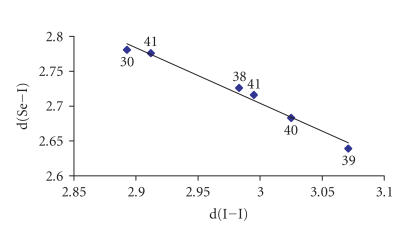
Graphical plot of d(Se−I) versus d(I−I) for [(*n*-PrSeU)I_2_] (30), (tzSeMe) · I_2_, (38) (tzSeMe = N-Methyl-thiazolidine-2(3H)-selone) [23], (btSeMe) · 2I_2_, (39) (btSeMe = N-Methyl-benzothiazole-2(3H)-selone) [23], {(L · I_2_) · (L_2_)^+^ · 2I^−^_3_} (40) (L = bis(N,N′-Dimethyl-imidazolidin-2-yl)-di-selenone) [24], (mbis) · 2I_2_, (41) (mbis = 1,1′-bis(3-Methyl-4-imidazolin-2-selenone)methane) [25].

[(*n*-PrSeU)I_2_] (30) was found to be a charge transfer complex with an Se−I bond. The I−I interatomic distance of 2.8928(10) Å is longer than that in either the gas phase (2.677 Å) or crystalline diiodine (2.717 Å at 110 K) presumably owing to the Se ⋯ I interaction. It is, however, the shortest such distance measured for a diiodine-selenoamide complexes suggesting a minimal perturbation resulting from the Se ⋯ I contact, which is the longest measured thus far [7]. The I−I bond order of 0.547 calculated for [(*n*-PrSeU)I_2_] from Pauling's equation d(I−I) = d_0_ −0.85·log(*n*) (1) (where d is I−I interatomic distance of the adduct, d_0_ is the I−I bond distance of gas phase I_2_ (2.67 Å) and *n* is the I−I bond order) [18] is the highest such bond order for selenoamide-diiodine complexes. All these data are consistent with a weak Se ⋯ I interaction, the weakest ever found. According to Bigoli et al classification, complex (30) is classified in the B type of adduct. It is interesting to note that the corresponding [(PTU)I_2_] complex forms a weaker ct complex, with an I−I bond order of 0.65*e* [3] compared to the 0.547*e* found for [(*n*-Pr−SeU)I_2_] which implies a weaker S ⋯ I interaction. With the same classification the former complex is of A type, while the latter of B type. Interestingly, there is a linear correlation between the Se ⋯ I and I−I distances (see Table 1 and Figure 2).

**Scheme 15:**
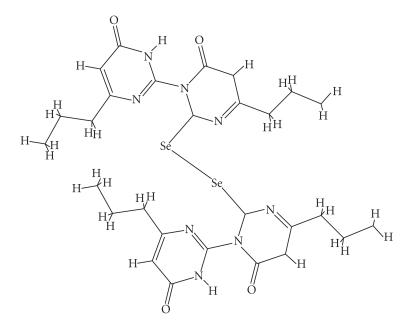
[N-(6-*n*-Pr-4-pyrimidone)(6-*n*-Pr-SeU)_2_](32).

The diselenides [N-(6-Et-4-pyrimidone)(6-Et-SeU)_2_] (31) and [N-(6-*n*-Pr-4-pyrimidone)(6-*n*-Pr−SeU)_2_] (32) (Scheme 15) were produced upon recrystallization of [(*n*-PrSeU)I_2_] (30) and [(*n*-EtSeU)I_2_] (33) from acetone, as oxidation products. On the other hand, deselenation with the formation of 6-*n*-propyl-2-uracil (*n*-Pr−U) (34) was observed when (30) was recrystallized from methanol/acetonitrile solutions [7].

**Scheme 16:**
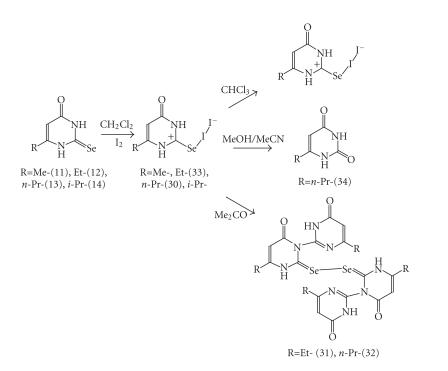


In conclusion, while 6-alkyl-2-selenouracil compounds (RSeU) (Scheme 16) are stable in various solvents, including water and other polar or nonpolar solvents, “spoke” ct complexes of formulae [(RSeU)I_2_] are formed in dichloromethane solutions, but are unstable in methanol/acetonitrile and/or acetone solutions (Scheme 16). [(RSeU)I_2_] is transformed to 6-alkyl-2-uracil in methanolic/acetonitrile solutions (Scheme 16). Upon recrystallization of the compound in acetone the diselenides containing also a covalent C−N bond with an adjacent PTU molecule are formed possibly through the formation of a substituted selenouracil as indicated by ^1^H, ^13^C NMR spectra, and ESI-MS spectra. The whole process may be hydrolytic (Scheme 16).

### Synthesis of amide-diiodine complexes

**Scheme 17:**
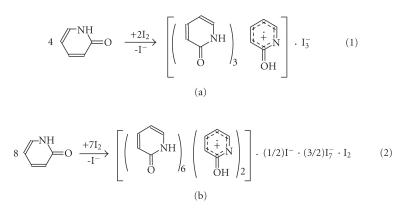


The reaction of 2-pyridone (PYOH) (15) with diiodine in a molar ratio of 2 : 1 and 1 : 2, respectively, resulted to the formation of {(PYOH)_3_[(PYOH)]^+^I^−^_3_} (35) and {(PYOH)_6_ · [(PYOH)_2_]^2+^ · ((1/2)I^−^) · ((3/2)I^−^_7_) · (I_2_)} (36) complexes (Scheme 17). The reactions were carried out in dichloromethane solutions.

The reaction of 2-pyrimidone (PMOH^+^_2_Cl^−^) (15) with diiodine in a molar ratio of 1 : 1 resulted to the formation of {[LOH]^+^Cl^−^I_2_} (37) complex.

**Figure 3:**
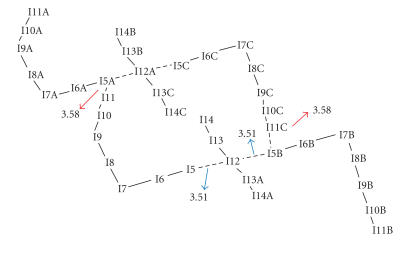
Polyiodine network established by weak halogen-halogen interactions, in the distance range 3.51 Å–3.58 Å, between I^−^_7_ and I_2_ ⋯ I^−^ ⋯ I_2_ ions in the {(PYOH)_6_ · [(PYOH)_2_]^2+^ · ((1/2)I^−^) · ((3/2)I^−^_7_) · (I_2_)}.

In case of complex (36) the counter anions form a polyiodine network. Figure 3 shows the polyiodine network established by weak halogen-halogen interactions, in the distance range 3.51 Å–3.58 Å, between I^−^_7_ and I_2_ ⋯ I^−^ ⋯ I_2_ ions forming an infinite chain.

In conclusion, structures containing polyiodide anions with cationic aromatic ligands as counterparts of formulae {[(L)(HL^+^)] · (I^−^_n_)} are known to be synthesized by the treatment of the appropriate amide with HI [28–30]. In contrast, the complexes with PYOH, in the present case, were formed by the direct reaction of 2-hydroxypyridine with diiodine in a molar ratio of 2 : 1 and 1 : 2. This is a redox reaction, where 2-hydroxy-pyridine firstly is oxidized to pyridinone-2 radical cation. In the case of 2-hydroxy-pyridine, however, peroxide structures are not formed like disulfides in the case of PYSH. Polyiodide anions are simultaneously produced in this case. This should be a consequence of redox differences between −SH and OH groups and may be proven a useful pathway for the synthesis of polyiodide materials.

## Figures and Tables

**Table 1:** Selected bond distances and angles of spoke and extended ct complexes.

Complex	I−I(I_2_) (Å)	I−I(S) (Å)	I−S (Å)	I−I−S (∘)	N−C−S−I−(∘)	*n* (e)* bond order	Type

[(BZT)I_2_] (17)	—	3.077(2)	2.728(6)	174.18(14)	166.40	0.33	C
[(PTU)I_2_] (18)	—	2.826(1)	2.780(1)	175.85(2)	−95.93	0.65	A
[(CBZT)I_2_] (19)	—	2.920(1)	2.633(1)	173.78(4)	167.88	0.51	B
[(NMBZT)I_2_] (20)	—	2.7912(1)	2.808(3)	176.94(7)	172.90	0.72	A
[(BZT)I_2_I_2_] (21)	2.7504(18)	2.969(2)	2.587(5)	177.78(13)	174.71	0.44	B
[(BZIM)I_2_I_2_H_2_O] (22)	2.767(3)	2.989(2)	2.571(6)	176.76(14)	2.95	0.42	B

*The I−I bond order calculated from Pauling's equation d(I−I) = d_0_ − 0.85 · log(*n*) (where d is I−I interatomic distance of the adduct, d_0_ is the I−I bond distance of gas phase I_2_ (2.67 Å), and *n* is the I−I bond order) [18].
